# Auxin polar transport in stamen formation and development: how many actors?

**DOI:** 10.3389/fpls.2014.00333

**Published:** 2014-07-16

**Authors:** Maura Cardarelli, Valentina Cecchetti

**Affiliations:** ^1^Istituto di Biologia, Medicina Molecolare e Nanotecnologie, CNR, Sapienza Università di RomaRome, Italy; ^2^Dipartimento di Biologia e Biotecnologie, Sapienza Università di RomaRome, Italy

**Keywords:** stamen development, auxin transport, Arabidopsis, dicots, monocots

## Abstract

In flowering plants, proper development of stamens, the male reproductive organs, is required for successful sexual reproduction. In *Arabidopsis thaliana* normally six stamen primordia arise in the third whorl of floral organs and subsequently differentiate into stamen filaments and anthers, where male meiosis occurs, thus ending the early developmental phase. This early phase is followed by a late developmental phase, which consists of a rapid elongation of stamen filaments coordinated with anther dehiscence and pollen maturation, and terminates with mature pollen grain release at anthesis. Increasing evidence suggests that auxin transport is necessary for both early and late phases of stamen development. It has been shown that different members of PIN (PIN-FORMED) family are involved in the early phase, whereas members of both PIN and P-glycoproteins of the ABCB (PGP) transporter families are required during the late developmental phase. In this review we provide an overview of the increasing knowledge on auxin transporters involved in Arabidopsis stamen formation and development and we discuss their role and functional conservation across plant species.

## Introduction

Stamens are the male reproductive structure of flowers and their function is to produce pollen grains, which house male gametes, and to release them at flower opening to allow plant reproduction. Male fertility results from sequential developmental events that involve an early phase of stamen formation and morphogenesis and a late phase that consists of pollen grain maturation, stamen filament elongation and anther dehiscence. In autogamous plants, such as *Arabidopsis thaliana*, stamen growth should be coordinated to pistil development to allow self-pollination. Alterations in stamen development lead to male sterile plants that can be utilized to generate hybrids useful for agronomic practice.

The plant hormone auxin, which is predominantly represented by indole-3-acetic acid (IAA), contributes to all aspects of plant development mainly through its differential distribution within plant tissues. Auxin concentration controls the expression of hundreds of genes by the ubiquitin-mediated pathway based on the interplay of two classes of transcription factors, auxin-response factors (ARFs) and Aux/IAA repressors (reviewed in Parry and Estelle, 2006). Local auxin concentration is the combined result of auxin biosynthesis and transport. The key enzymes involved in the main auxin biosynthetic pathway belong to YUC family of flavin monooxygenases -which consists of 11 *YUC* genes in Arabidopsis (reviewed in Zhao, 2012) and show distinct but partially overlapping expression patterns during diverse developmental programs. Differential distribution of synthesized auxin is mainly achieved by a polar cell-to-cell transport system (PAT), and this is unusual among phytohormones, given that polar transport has not been detected for any other signaling molecules (Benjamins and Scheres, 2008; Zažímalová et al., 2010). The protonated form IAAH can either enter cells passively due to the low pH in cell walls or can be pumped into cells by influx carrier of the AUX/LAX family (Swarup and Péret, 2012). In contrast, due to the higher pH in the cytosol, auxin cannot cross the membrane and the anion IAA^−^ can only exit from cells by efflux carriers. Proteins of the PIN-FORMED (PIN) family are the main group of efflux carriers with a polar cellular distribution (Gälweiler et al., 1998; Friml et al., 2003; Paponov et al., 2005; Friml, 2010). PINs carriers can be divided in a large-loop and a short-loop subgroups. Large-loop PINs (PIN1, PIN2, PIN3, PIN4, and PIN7), characterized by a large hydrophilic loop, localize to the plasma membrane, and direct auxin transport across those membranes where they are localized. Short-loop PINs (PIN5, PIN6, and PIN8) are devoided of the large hydrophilic loop (Paponov et al., 2005), and are not recruited to the plasma membrane but are proposed to regulate auxin homeostasis between the cytoplasm and endoplasmic reticulum (ER) (Mravec et al., 2009; Wabnik et al., 2011). Recently, a family of seven PIN-LIKES proteins (PILS) localized in the ER has been described as regulators of intracellular auxin homeostasis (Barbez et al., 2012). In addition to the PINs, the ABCB/multidrug resistance/P-glycoproteins (ABCB/MDR/PGP) are ATP Binding Cassette subfamily B (ABCB) transporters associated with polar auxin transport mainly in auxin efflux. Of the subfamily, at least five members have been reported to mediate cellular transport of auxin (or auxin derivatives) coordinately with PIN proteins (Blakeslee et al., 2005; Titapiwatanakun et al., 2008; Zažímalová et al., 2010; Peer et al., 2011).

Different research findings have provided a role for PAT in early and late phases of stamen development. In this review, we discuss the present knowledge on the contribution of auxin transport to Arabidopsis stamen formation and development and functional conservation of different auxin transporters in other species.

## Stamen structure

**Figure 1:**
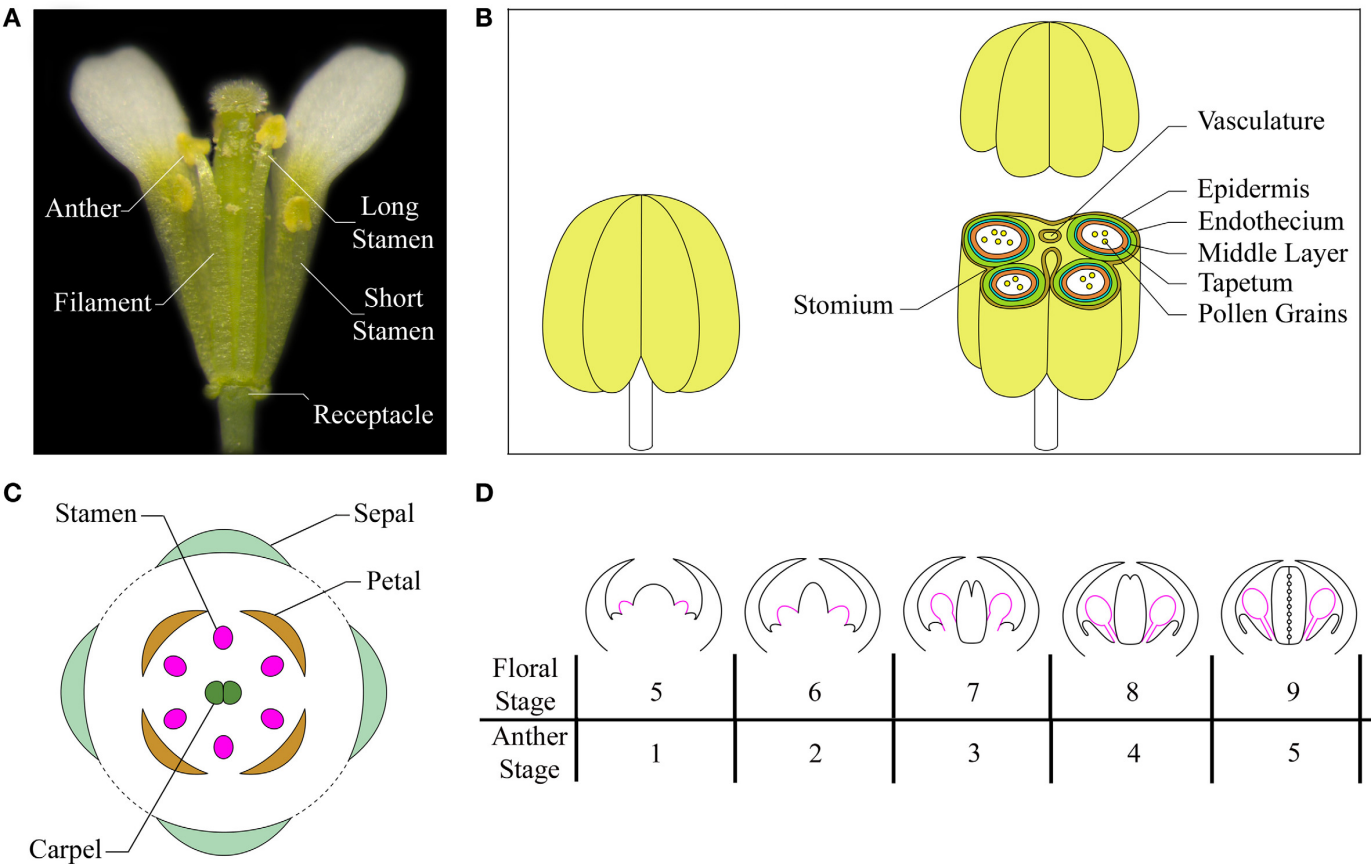
**The Arabidopsis stamen. (A)** A mature flower at anthesis showing short and long stamens (with the removal of some petals and sepals for visualization). **(B)** Cartoon of a stamen at a stage after meiosis (left) and of the stamen transverse section, at the level of the anther, with differentiated tissues indicated in different colors (right). Based on Sanders et al. (2000), Jia et al. (2008), Smyth (2010). **(C)** A diagram showing different floral organs with stamens depicted in pink. Based on Bowman (1994), Whipple et al. (2004). **(D)** Schematic representation of early development of stamens in Arabidopsis flowers at floral stages from 5 to 9/anther stages from 1 to 5. Stamens are depicted in pink. Based on Ito et al. (2007), Alvarez-Buylla et al. (2010).

Arabidopsis has four long and two short stamens—collectively termed androecium—each of which consists of two morphologically distinct parts: the filament and the anther. The filament, which serves as a conduit for water and nutrients, has a simple radialized structure with a single vascular strand. It provides structural support to the anther and anchors the stamen to the receptacle, the stem to which the floral organs are attached (Figure 1A). The anther contains the reproductive and non-reproductive tissues that contribute to pollen maturation, protection, and release, so that the self-pollination process can occur into the flower. The anther has a bilaterally symmetrical four lobed structure and each lobe contains a microsporangium (pollen sac) in which pollen develops in chambers known as locules. The anther lobes are laterally attached to a vascular bundle by a contiguous parenchymatous tissue called connective, and the vascular bundle is continuous with the vasculature of the filament (Sanders et al., 1999; Ma, 2005; Murmu et al., 2010). A transverse section of a differentiated anther is butterfly-shaped and each of the four locules is surrounded by four distinct, concentric layers of cells with special tasks in stamen development: an inner wall layer called the tapetum—adjacent to the sporogenous cells—necessary for pollen nourishment and development; the thin layer termed middle layer, whose function has also been associated to pollen development; the endothecium, a subepidermal tissue required for anther opening and pollen release; and the outer epidermis, that covers and protects all the microsporangia (Figure 1B) (Esau, 1977; Pacini et al., 1985; Goldberg et al., 1993; Scott et al., 2004; Wilson et al., 2011).

## Stamen development

Flower morphogenesis begins when the inflorescence meristem produces floral meristems on its flanks. Floral organ primordia arise from floral meristems in a precise number and are arranged in concentric whorls forming the basic flower structure with four types of organs: sepals, petals, stamens, and carpels (Figure 1C). Floral organ primordia identity is determined by the combined action of four classes of floral organ identity genes (A, B, C, and E), according to the genetic ABCE model (reviewed in Wellmer et al., 2013). Stamen formation occurs in the third whorl due to the combination of BCE class gene activity.

**Figure 2:**
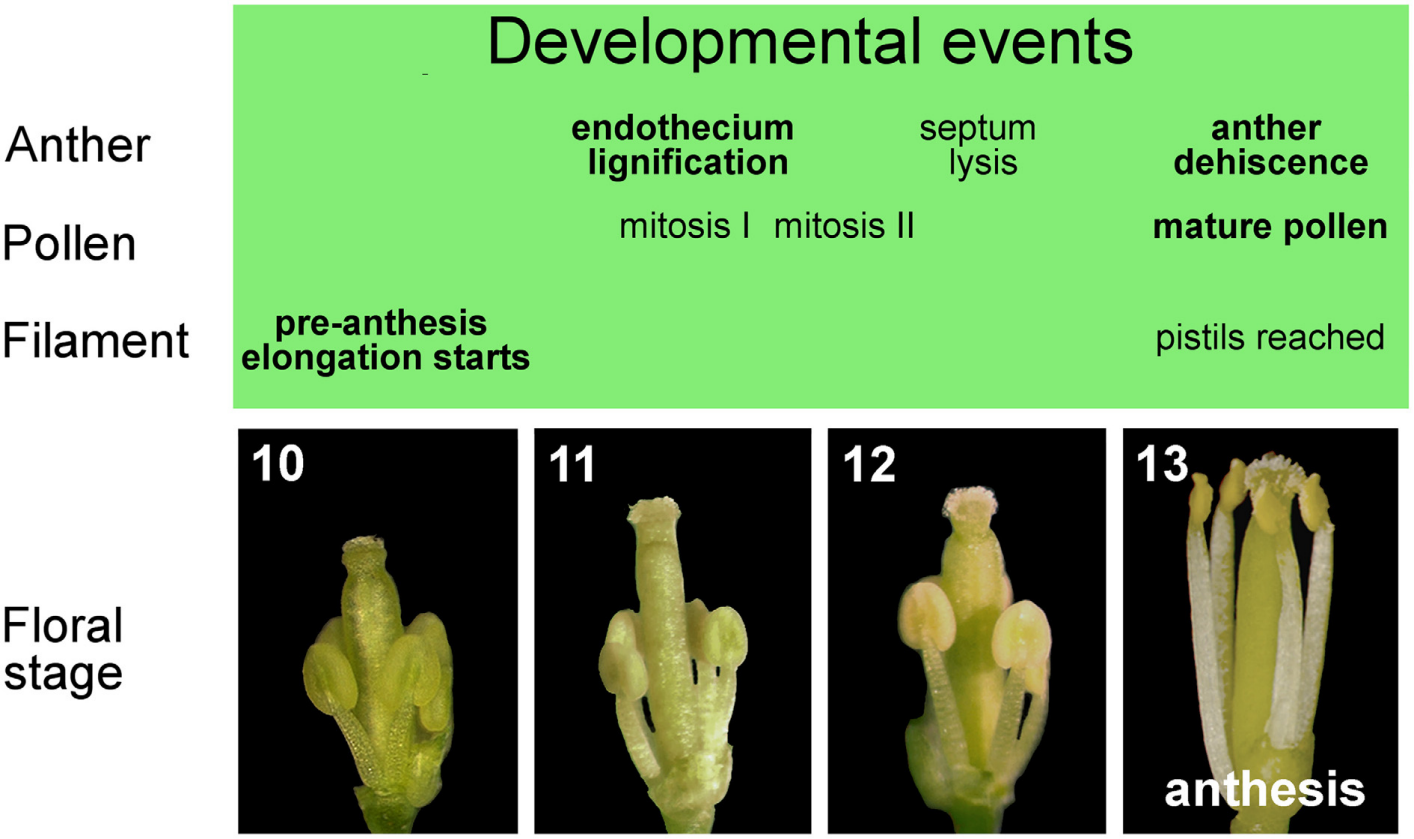
**Late development of stamens in flower buds from stages 10 to 13**. Main events in the development of anther, pollen, and filament occurring in stamens during late flower development.

The entire stamen developmental process in Arabidopsis is divided into an early and a late phase. During the early phase, primordia arise and histospecification, morphogenesis and microsporogenesis occur (Figure 1D). During the late phase, microspores differentiate into pollen grains, stamen filaments elongate to reach the pistil, anthers enlarge and a tissue degeneration process occurs inside the anther, leading to anther dehiscence and pollen grain release at flower opening (Figure 2) (Goldberg et al., 1993; Cecchetti et al., 2008; Feng and Dickinson, 2010).

In Arabidopsis, six stamen primordia appear, due to divisions in the L1, L2, and L3 layers of the floral meristem during stage 5 of flower development (as defined by Smyth et al., 1990; Bowman, 1994) or anther stage 1 (as defined by Sanders et al., 1999), with the long stamens primordia arising first (Figure 1D). Stamen primordia appear at the same time as the petal primordia, after the appearance of sepal, but before gynoecium primordia. From floral stage 5 to 9 (or /anther stage 1–5) divisions in the L1 layer form the stamen epidermis, while L3 cells divide to form connective and vasculature tissues. Periclinal divisions of L2 cells, called archesporial cells, form primary parietal and primary sporogenous cells that give rise to the four radially symmetrical microsporangia. By floral stage 7/anther stage 3, the regions that will give rise to the filament and the anther become distinct. At floral stage 8/anther stage 4 two larger abaxial anther locules and two smaller adaxial pair are visible and are separated by the connective tissue. Next, the primary parietal cells give rise to the three anther wall layers (tapetum, middle layer and endothecium), and the primary sporogenous cells develop into microspore mother cells (floral stage 8/anther stage 4). Mother cells go through meiosis to form a microspore tetrad, surrounded by a callose wall that isolates meiotic cells (floral stage 9/anther stage 5). Most of early stamen growth occurs at floral stage 9 mainly in the anther region, while only 20% of the total stamen length is due to the filament. By stage 9, the anther shows the characteristic four-lobed morphology and anther morphogenesis is complete (Figure 1D).

From floral stage 10 to 13, late development occurs, which consists of three different developmental programs: pollen maturation, anther dehiscence and filament elongation. At the beginning of late development at floral stage 10/anther stage 8, the callose wall surrounding the tetrads degenerates, leading to the release of individual microspores into the anther locules. The microspores then generate an exine wall, and become vacuolated at floral stage 10/anther stage 9. The first mitotic division of microspores occurs at floral stage 11/anther stage 10 and gives rise to bicellular pollen grains, while the second pollen mitotic division occurs at floral/anther stage 12, resulting in tricellular haploid pollen grains that are capable of germinating at anthesis (floral/anther stage 13) (Figure 2). The main developmental phases of anther dehiscence are: degeneration of the middle layer and tapetum at floral stage 11/anther stage 10; expansion of the endothecial layer followed by deposition of fibrous bands (wall thickenings) in endothecial (and connective) cells at floral/anther stage 11; degeneration of the septum—the cells separating the two locules—which generates a bilocular anther at floral/anther stage 12. The breakage of the stomium, a group of specialized epidermal cells, is the final event and occurs at anthesis—floral stage 13/anther stage 12. The pre-anthesis growth of the stamen filament takes place from floral stage 10 to anthesis and is particularly rapid from stage 12–13 due to cell elongation. At floral stage 13 (Figure 2), when flower opens, with petals bent outwards, stamens have reached the pistil, anther opening occurs, and the filaments continue to extend to allow the subsequent pollen deposition on the receptive stigma at floral stage 14 (Smyth et al., 1990; Bowman, 1994; Sanders et al., 1999; Scott et al., 2004; Cecchetti et al., 2008).

## Auxin transport and the early phase of stamen development

### Stamen primordia formation

In addition to floral meristem initiation, auxin controls the early phase of floral organ primordia formation and morphogenesis. Local auxin biosynthesis, transport, and signaling are all critical for stamen (and floral organs) initiation as a reduction in stamen number is observed in different classes of mutants. Single *yuc4* mutant flowers -defective in the auxin biosynthetic gene *YUC4*- show one or two stamens, and few floral organs (Ståldal et al., 2012), while the *yuc1yuc4* double mutant flowers show only stamen-like structures and few outer whorl organs. This latter phenotype is rescued by the expression of the bacterial auxin biosynthetic gene *iaaM* under the control of the *YUC1* promoter (Cheng et al., 2006; Krizek, 2011). Mutants defective in different *AUXIN RESPONSE FACTOR* (*ARFs*) genes such as *ARF3/ETTIN* or *ARF5/MONOPTEROS*, show flowers with reduced number of stamens, together with increased number of sepals and petals and abnormal gynoecia (Sessions et al., 1997), or few flowers with reduced number of stamens, together with less petals and a single carpel respectively (Przemeck et al., 1996). A reduced number of stamens, as well as no floral buds or flowers with no stamens, were first related to a decreased auxin transport, in plants cultured in the presence of different auxin transport inhibitors such as 1-naphthylphthalamic acid (NPA) (Okada et al., 1991; Reinhardt et al., 2000) and 2,3,5-triiodobenzoic acid (TIBA) (Thomson et al., 1973). These phenotypes strongly resembled those of mutants defective in the auxin polar transporter PIN1: the *pin-1* strong mutant was unable to form flowers (Okada et al., 1991; Bennett et al., 1995; Gälweiler et al., 1998), while few flowers that fail to form stamens or flowers with a reduced number of stamens were observed in weaker alleles such as *pin-3*, *pin-4*, and *pin*-5, respectively. Similarly *pid* mutants, defective in the *PINOID (PID)* protein kinase that controls PIN1 polarity, show flowers with a range of defects that can include a pin-shaped inflorescence or alterations in stamen number (Bennett et al., 1995). In addition, *mab4* mutants, defective in the enhancer of *PID, MACCHI-BOU 4* (*MAB4*), show flowers with a reduced number of stamens (Treml et al., 2005; Furutani et al., 2007). Indeed, *PIN1* and *PID* expression is high at the site of incipient stamens in young flowers (Christensen et al., 2000; Reinhardt et al., 2003). Other members of the PIN family seem also to be involved in stamen primordia formation; for example, *pin3pin7* double mutant flowers show no stamens, together with bear fused petals, and occasionally no sepals, suggesting a role for both PINs in stamen formation (Benková et al., 2003). On the other hand PIN6, one member of the short-loop PINs, promotes short stamen primordia formation, as the loss-of-function mutant *pin6*–*2* lacks one or both short stamens (Bender et al., 2013). Benková et al. (2003) analyzed the auxin distribution during floral organ formation by monitoring the expression gradients of the auxin responsive element DR5, an indirect reporter of auxin accumulation in plant cells (Ulmasov et al., 1997; Michniewicz et al., 2007); DR5 expression reaches a maximum at the tip of primordia in all floral organs. Based on the correlation between PIN1 localization, DR5 gradients, and primordia formation, as well as on the interference in DR5 activity exerted by auxin efflux inhibitors, they proposed a model for all developing floral primordia. According to their model, floral organ primordia formation is dependent on apical auxin transport mediated by PIN-dependent efflux, which supplies auxin to the tip through the outer layer L1; in the inner layers, PINs transport auxin away from the tip through the primordium interior, giving rise to the provascular strands. This general model can also be applied to stamens, since van Mourik et al. (2012) showed that when stamen primordia arise—at the transition of stage 4 to 5—DR5 signal peaks in a small group of cells in the central region of the floral meristem, where initiation of the 4 long stamen primordia occurs. DR5 signal is observed in L1 cells, in the cell layers below where provasculature is formed and in deeper layers where the provasculature of both petals and stamens develop. In addition, based on a simulation study of auxin transport through a growing floral meristem, van Mourik et al. (2012) proposed that sepals are initiated by the auxin maxima forming at the tip during early meristem outgrowth. Then the sepals direct the positions of the smaller auxin maxima associated with the petal, stamen, and carpel anlagens—the initial clustering of cells from which primordia develop.

PAT is also involved in the formation of boundaries between organ primordia, by regulating the expression of the *CUC1* and *CUC2* genes. The role of these genes is to inhibit the growth of cells at the boundaries between primordia to avoid organ fusion. As *PID* and *CUC* expression domains are overlapping in the boundary of cotyledon primordia, it has been suggested that PID, by promoting auxin transport, reduces the level of auxin at the boundary and increases it in the primordia during cotyledon formation (Furutani et al., 2004). This model can be also applied to stamens, as *CUC1* is expressed in boundaries between stamen primordia at stage 5–6 of floral development (Takada et al., 2001). In agreement, in *pid* (Bennett et al., 1995) and *mab4-1* (Furutani et al., 2007) mutant flowers, bifid stamens are frequently observed, with two filaments fused partly or completely along their length.

Apparently, ABCB/PGP transporters have no effect on the formation of stamen primordia, as mutants defective in single *ABCB/PGP* genes as well as the *abcb1abcb19/pgp1pgp19* double mutants are not altered in the number of stamens. However, analysis of the triple *pin1pgp1pgp19* mutant—which lacks *PIN1* in addition to *PGP1* and *PGP19*—show partial rescue of the *pin1* mutant phenotype and formation of few flowers (Blakeslee et al., 2007). In contrast the double *pin1pgp19* is unable to form flowers. This suggests that the loss of *ABCB1/PGP1* is epistatic to *PIN1* in floral meristems, and that the partial restoration of flower formation could be due to ectopic auxin accumulation in the floral meristem. This effect is only exerted by *ABCB1/PGP1* loss in the *pin1* background and suggests that *ABCB1*, but not *ABCB19*, contribute to floral organ primordial formation (Blakeslee et al., 2007). The role of auxin influx carriers in stamen primordia formation has not yet been clarified. It has been shown that *aux1* single mutant, defective in the influx carrier AUX1, show normal flower development without reduction in the number of stamens. However, mutations in *AUX1* and in its paralogs *LAX1*, *LAX2*, and *LAX3* lead to quadruple mutant flowers with an abnormal structure, showing defects in floral organ number and positioning (Bainbridge et al., 2008).

**Figure 3:**
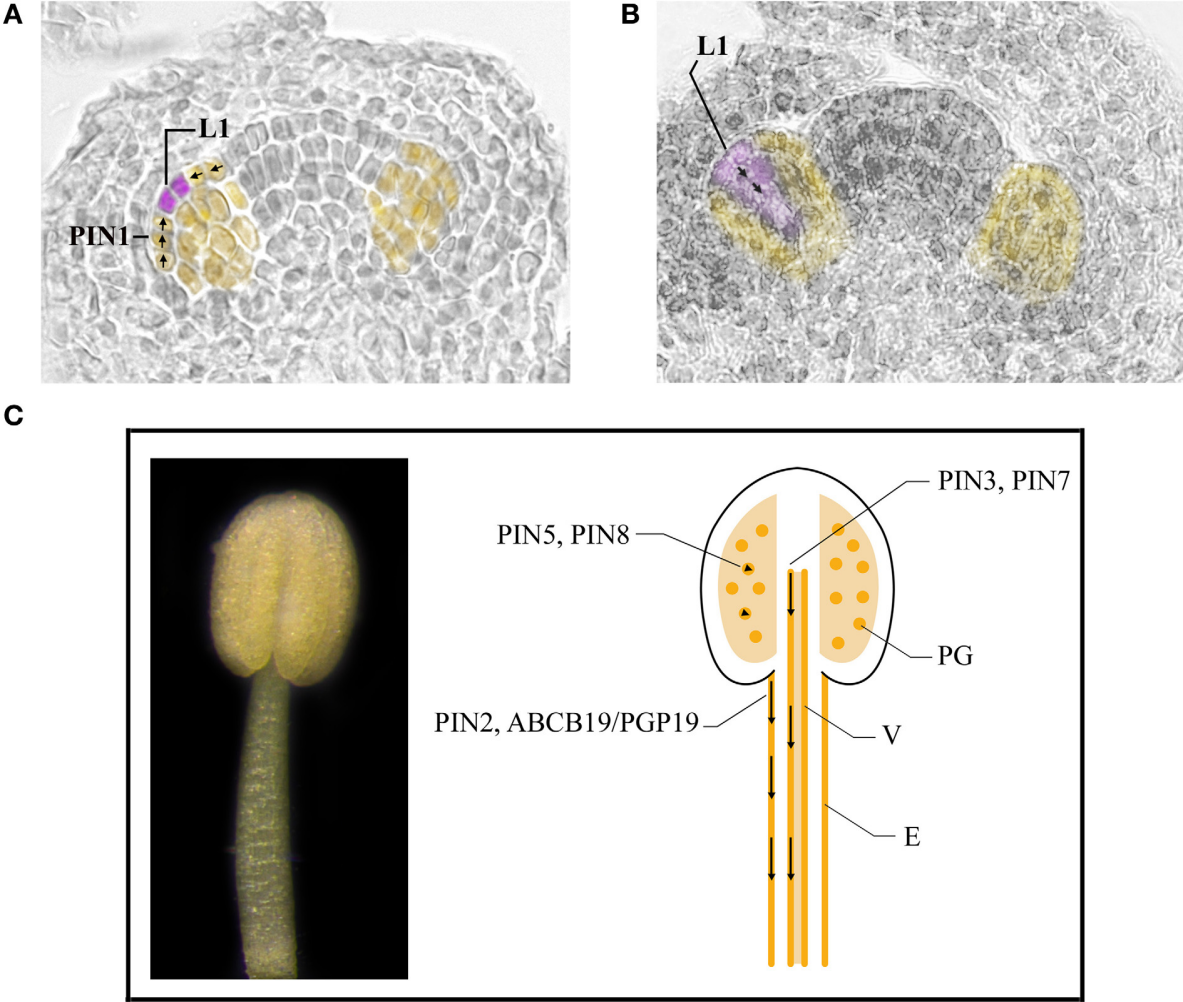
**Auxin transport in stamens during early and late developmental stages. (A,B)** Stamen primordia (yellow) at stage 5 **(A)** and 6 **(B)** of flower development. Places of auxin accumulation are depicted in purple. Presumptive routes of auxin transport are depicted by black arrows. **(C)** A stamen at stage 11 of flower development (left). Cartoon of a longitudinal section of a stamen at stage 11 of flower development (right). Basipetal auxin transport in the stamen filament is depicted by black arrows in epidermal cells and vascular tissue. Arrowheads indicate regulation of auxin homeostasis inside pollen grains. E, epidermal cells; L1, L1 layer; PG, pollen grains; V, vascular tissue.

Taken together this data (summarized in Table 1) suggest that auxin, synthesized by *YUC* genes in young floral buds, is first transported directionally toward the tip of the primordium, where it reaches a maximum necessary for stamen primordia formation and then is transported basipetally to the interior of the primordium as shown in the model in Figures 3A,B. Auxin transport is mainly carried out by PIN1, with a contribution of PIN3 and PIN7 in the 4 long stamens formation, which still needs to be clearly defined. Further studies are needed to determine the role of ABCB1 which functions primarily in minimizing apoplastic reflux in apical tissues with high auxin (Geisler and Murphy, 2006) and that of influx carriers AUX1, LAX1, LAX2, and LAX3, which could concentrate auxin in the cytoplasm of cells of the L1 layer, thus preventing auxin diffusion from the L1 (Bainbridge et al., 2008).

**Table 1:** **Summary of the role in Arabidopsis stamen development of auxin-related genes**.

**Gene(s)**	**Mutant(s)**	**Phenotype(s)**	**Gene function**	**Stamen development stages**	**Reference(s)**
*PIN1*	*pin-3*	No stamens	Polar auxin efflux	Long stamen primordia formation	Bennett et al., 1995
	*pin-4*	No stamens			
	*pin-5*	Few stamens			
*PIN3*	*pin3-5pin7-1* double mutant	No stamens	Polar auxin efflux	Long stamen primordia formation	Benková et al., 2003
*PIN7*					
*PIN6*	*pin6****–****2*	One or both short stamens missing	Intra-cellular auxin homeostasis	Short stamen primordia formation	Bender et al., 2013
*PINOID*	*pid-1*	Few stamens	Ser/Thr	Stamen primordia formation	Bennett et al., 1995
	*pid-2*	Few stamens	Kinase, regulator of auxin efflux	Stamen morphogenesis	
	*pid-8*	Abnormal anthers			
*MACCHI-BOU 4*	*mab4-1*	Few stamens	Enhancer of *PINOID*	Stamen primordia formation	Furutani et al., 2007
		Abnormal anthers		Stamen morphogenesis	
*PIN2*	*eir1-4*	Shorter filaments	Polar auxin efflux	Late stamen development	Kim et al., 2013
*AP-2*	*ap2m-1*	Shorter filaments	Clathrin-mediated endocytosis	Late stamen development	Kim et al., 2013
	*ap2m-2*	Defective pollen grains	Regulator of PIN localization		
		Altered pollen tube growth			
*PIN8*	*pin8-1*	Aborted or misshaped pollen grains	Intra-cellular auxin homeostasis	Late stamen development	Ding et al., 2012
*PIN5*	*pin5-5*	Defective pollen grains	Intra-cellular auxin homeostasis	Late stamen development	Ding et al., 2012
*ABCB1*	*pgp1pgp19* double mutant	Shorter filaments	ABC transporters, Auxin efflux	Late stamen development	Noh et al., 2001
*ABCB19*		Early dehiscent anthers	Mediators		Cecchetti et al., 2008
*YUC4*	*yuc4-1*	Few stamens	Auxin biosynthesis	Stamen primordia formation	Ståldal et al., 2012
*YUC1*	*yuc1yuc4* double mutant	Stamen-like structures			Cheng et al., 2006
*YUC2*	*yuc2yuc6* double mutant	No pollen grains	Auxin biosynthesis	Late stamen development	Cheng et al., 2006
*YUC6*		No stamen elongation			
		Altered anther dehiscence			
*ARF5/ MONOPTEROS*	*mp^T370^*	Few stamens	Auxin response	Stamen primordia formation	Przemeck et al., 1996
	*mp^G92^* gain-of-function	Few stamens	Transcription factor	Late stamen development	Przemeck et al., 1996
	*mp^abn^*	Indehiscent anthers			Garrett et al., 2012
*ARF3/ETTIN*	*ett-1*	Few stamens	Auxin response	Stamen primordia formation	Sessions et al., 1997
			Transcription factor		
*ARF17*	*arf17*	Altered pollen wall patterning	Auxin response	Late stamen development	Yang et al., 2013
		Altered pollen tube growth	Transcription factor		
*ARF2*	*mnt*	Altered stamen length	Auxin response	Late stamen development	Schruff et al., 2006
*ARF1*	*arf2-8*	Shorter filaments	Transcription factor		Ellis et al., 2005
	*arf1-5arf2-8* double mutant	Shorter filaments			Ellis et al., 2005
*ARF6*	*arf6-2arf8-3* double mutant	Short filaments Indehiscent anthers	Auxin response	Late stamen development	Nagpal et al., 2005
*ARF8*			Transcription factor		
*TIR1*	*tir1afb1afb2afb3* quad mutant	Shorter filaments	Auxin receptors	Late stamen development	Cecchetti et al., 2008
*AFB1*		Early dehiscent anthers			
*AFB2*		Precocious pollen maturation			
*AFB3*					
*IDD14*	*idd* triple mutant	Asynchronous elongation of stamen filaments and styles	Transcription factors	Late stamen development	Cui et al., 2013
*IDD15*			Regulators of the expression of *YUC5* and *PIN1*		
*IDD16*					

### Stamen morphogenesis

Auxin is synthesized also in the subsequent stages of stamen morphogenesis when different auxin biosynthetic genes such as *YUC2* and *YUC6* are expressed. Indeed, detectable auxin levels, as measured by gas chromatography analysis, can be observed in anthers at stages 8 and 9, although DR5 activity is visible only later, at the beginning of late development (Cheng et al., 2006; Cecchetti et al., 2008).

Scattered evidence suggests the involvement of auxin transport in stamen morphogenesis. As described by Bennett et al. (1995) *pid* anthers are often abnormal, with locules occurring in various numbers and shapes while *pid* pollen is fertile. This data suggests that auxin transporters, which are target of PID phosphorylation are required for stamen morphogenesis but are not involved in microspore formation and development (see below). In addition, it has been recently shown that *PIN6* is expressed in anthers at stage 8 of flower development/anther stage 4, as detected by GUS staining of proPIN6:GUS flowers. This would suggest that PIN6, in addition to its role in short stamen formation (see above), could also be necessary for the formation of long stamens (Bender et al., 2013).

## Auxin transport and different processes of late stamen development

Auxin is a key regulator of the developmental processes occurring late in stamen development: anther dehiscence, pollen maturation, and pre-anthesis filament elongation.

Intense *DR5:GUS* expression has been observed in stamens at different stages of late development (Aloni et al., 2006; Feng et al., 2006). At the end of meiosis (floral stage 10/anther stage 9) DR5 activity is observed in anther sporophytic tissues surrounding the locules (tapetum, middle layer, and endothecium), as well as in microspores and in vascular cells of the anther and the filament. At floral stage/anther stage 11 DR5 activity was mainly detectable in the remnants of tapetum, in immature pollen grains, and in the anther-filament vasculature, whereas it is no longer detectable at floral stage/anther stage 12 after septum lysis (Cecchetti et al., 2008). By measuring IAA concentration in Arabidopsis anthers at various developmental stages it was shown that DR5 activity corresponds to changes in free IAA concentrations (Cecchetti et al., 2013). Further, all three developmental processes are severely altered in *tir1afb* multiple mutants, which lack the auxin receptors TIR1 and AFB (Cecchetti et al., 2008). Accordingly, *ARF* mutants show stamen development defects: *arf6arf8* double mutants fail to elongate stamen filaments at anthesis and show indehiscent anthers, while *arf2* single mutant flowers exhibit altered stamen length (Ellis et al., 2005; Nagpal et al., 2005; Schruff et al., 2006). Recently it has been shown that *mp*^abn^ the gain-of-function mutant of *ARF5/ MONOPTEROS*, shows indehiscent anthers (Garrett et al., 2012). In addition *arf17* single mutant flowers are defective specifically in pollen wall patterning and pollen tube growth (Yang et al., 2013). A peak in auxin concentration is observed at the beginning of late development, but auxin synthesis probably starts before, at premeiotic and meiotic stages, as suggested by the expression of *YUC2* and *YUC6* genes in stamens (Cecchetti et al., 2013). Accordingly, in *yuc2yuc6* double mutant flowers, no pollen grains are formed and stamen elongation does not occur, leading to sterile flowers, and indicating a block in late stamen development, which can in fact be rescued by expression of the bacterial auxin biosynthesis gene *iaaM* under the control of *YUC6* promoter (Cheng et al., 2006).

The observed auxin accumulation at the beginning of late development is not due to the transport of auxin through the filament to anthers. Indeed, blocking transport by NPA treatment of *DR5:GUS* flowers before late development (at premeiotic and meiotic stages), did not impair DR5 activity in stamens; similarly, in anthers severed at a premeiotic stage from *DR5:GUS* flower buds and matured *in vitro*, GUS staining was not altered and was comparable to control *DR5:GUS* anthers (Cecchetti et al., 2008). However, increasing evidence shows that PAT not only is fundamental for stamen filament elongation, but also has a role in pollen development and anther dehiscence. NPA-treated flowers showed shorter filaments if treated at floral stage/anther stage 11 when, as judged from DR5 activity, auxin has already built up in stamens (Cecchetti et al., 2008). Furthermore, *pgp1pgp19* double mutant flowers have reduced stamen filament elongation (Noh et al., 2001). Accordingly, *ABCB19/PGP19* is expressed in stamen epidermal cells, where it functions primarily in basipetal auxin transport from the stamen filament apical region to the basal side (Blakeslee et al., 2007; Titapiwatanakun and Murphy, 2009). Recently, a polar localization of PIN2 in the stamen filaments has been observed by Kim et al. (2013); *eir1-4* plants, which have a loss-of-function mutation in *PIN2*, showed defects in filament elongation and a reduced DR5 activity in anthers and in basal sides of the filaments. The authors also show that the shorter filament phenotype was observed in 30% of *eir1-4* flowers, compared to 90% of *ap2m-1* flowers, defective in the adaptor protein complex 2 (AP-2), which regulates polar distribution of PINs. This raises the possibility that auxin levels are also modulated by other PIN proteins in addition to PIN2 in stamens (Kim et al., 2013). Some indirect evidence in support to this hypothesis comes from studies on a family of nine early auxin-responsive genes, called *SMALL AUXIN UP RNAs* (*SAURs*). In particular, GUS staining was observed in the vascular tissues of *SAUR63:GUS* stamens and reduced expression of *SAUR63* causes a short stamen phenotype whereas lines overexpressing *SAUR63* showed long stamen filaments. Quite interestingly, when a *SAUR63:GUS* translational fusion construct was expressed in *pgp1pgp19* plants, their flowers had longer stamen filaments than *pgp1pgp19* flowers. In contrast, when *SAUR63:GUS* was expressed in *pin3-4pin7-2* double mutant plants, their filament length was comparable to that of *pin3-4pin7-2* double mutant flowers (Chae et al., 2012). Thus, *SAUR63* could promote auxin-induced growth of stamen filament during late development via PIN3 and PIN7, but not via ABCB1/PGP1 and ABCB19/PGP19, possibly acting on perivascular cells to direct auxin flux.

The involvement of auxin transport in pollen development mediated by short-loop PINs has been reported in different papers. According to transcriptomic data (Honys and Twell, 2004; Pina et al., 2005; Wang et al., 2008), *PIN8* and *PIN6* are expressed during pollen development at high and low levels, respectively and, in agreement, *pin8* mutation resulted in aborted or misshaped pollen grains, though at very low frequencies (Ding et al., 2012). Moreover, an antagonistic/compensatory activity of PIN8 and PIN5 has been suggested by Ding et al. (2012) who has showed that *pin5* loss of function mutants displayed the same percentage of defective pollen grains as *pin8*, whereas *pin5pin8* double mutants could rescue the pollen morphology defects observed in the single mutants. Some others transporters can be involved in pollen maturation and germination as suggested by transcriptomic data: *PILS5*, which encodes for a protein localized in the ER like PIN5, PIN6, and PIN8, is widely expressed during pollen development, from bicellular to mature pollen, as well as during pollen germination according to Dal Bosco et al. (2012b). In addition a low level of expression of the large-loop PIN genes, *PIN1*, *PIN2* and *PIN4* has been reported in mature pollen grains (Dal Bosco et al., 2012a; Kim et al., 2013). Because *ap2m-1* flowers have both an altered PINs polar distribution, and an impaired pollen germination, it is possible that specific polar PINs, might be involved in maintaining auxin homeostasis in pollen tube elongation (Kim et al., 2013). Further experiment should shed light on their molecular identity.

Little data has been reported on the role of auxin transporters on the anther dehiscence process. However, Cecchetti et al. (2008) showed that *pgp1pgp19* flowers, in addition to short stamen filaments, also exhibit a small percentage of early dehiscent anthers. On the other hand, treatment with NPA of flower buds at floral stage 11 resulted in 15–20% of indehiscent anthers at stage 13.

Taken together, this data, summarized in Table 1 indicates that the basipetal auxin transport from the filament apex to the basis is responsible for proper stamen filament elongation during late stamen development (Cecchetti et al., 2008; Titapiwatanakun and Murphy, 2009). Different transporters are involved in auxin transport in epidermal cells such as PIN2, ABCB19/PGP19 and ABCB1/PGP1 or in perivascular tissues such as PIN3 and PIN7 as shown in the model described in Figure 3C.

A main role in pollen maturation seems to be provided by ER-localized PINs, such as PIN8 and PIN5, and possibly PILS5. These proteins should be able to transport IAA from the cytoplasm to the ER, where enzymes involved in conjugation/hydrolysis and storage of IAA are accumulated, thus affecting auxin concentration. As it is known that developing and germinating pollen grains have high auxin levels, it is possible that the ER-localized PINs regulate the release of auxin from the internal stores in the ER to control pollen development and to drive auxin-mediated pollen tube elongation. Data on large-loop PINs suggests that auxin is also provided to pollen grains by polar transport. Two models have been proposed. Auxin could be transported to developing pollen grains from tapetum cells, which are known to accumulate auxin at the beginning of late development, as proposed by Aloni et al. (2006). Alternatively, according to Feng et al. (2006), auxin could be transported to developing pollen grains from the stamen filament.

In contrast, the current available data does not allow the construction of a model on auxin distribution in different tissues during the anther dehiscence process, but does suggests that auxin transport through ABCB1 and ABCB19 regulates the timing of anther dehiscence (Cecchetti et al., 2008).

Stamen and carpel development need to be coordinated to allow self-fertilization at anthesis. The *arf1* and *arf2* single mutants show defects in both stamen filament and in the length of style—the gynoecium portion, which connects the stigma with the ovary. These defects could be related to auxin transport, as auxin distribution seems to be finely regulated during late development in the style (Girin et al., 2011). Thus, it is possible that auxin transport during late floral stages allows the coordination of style and stamen elongation. Indeed, it has been recently shown that flowers from the triple mutant *idd*, defective in the *INDETERMINATE DOMAIN* (*IDD*) transcription factor genes *IDD14*, *IDD15*, and *IDD16*, exhibits infertile siliques resulted from the asynchronous elongation of stamen filaments and styles. The transcription of *AUX1*, *PIN1*, *ABCB1*, and *ABCB4* is severely reduced in the *idd* mutant, and the IDD proteins could directly bind to the promoter regions of *PIN1* to activate its expression (Cui et al., 2013).

## The role of auxin transport during early and late phases of stamen development in other species

Additional information is emerging regarding the role of auxin transport in early and late phases of stamen development in other angiosperms, mainly by determining the function of orthologs in other species of Arabidopsis genes already known to be involved in auxin synthesis, polar transport, and signal transduction. Summarized below are the most abundant information on PAT in dicots and monocots.

### Dicots

As described earlier for Arabidopsis, dicot flowers have in common arrangement in four whorls with an outermost whorl composed of sepals and the second one of petals. The third and the fourth whorls contain the reproductive organs, stamen, and carpel respectively.

The tomato (*Solanum lycopersicum*) floral system is distinguished from that of Arabidopsis as tomato flowers contain five sepals, alternating with five petals, five anti-sepalous stamens (stamen attachment is in line with the sepals) and two fused carpels are observed. Stamens are fused together to form a cylinder that surrounds the style.

Tomato has long served as a major model for fruit development and information on the role of auxin on stamen development mostly derives from experiments that were not directly focused on male reproductive organs. For example, downregulation of *SlIAA9*, a member of the Aux/IAA transcription factor gene family encoding a negative auxin response regulator, gives rise to a parthenocarpic fruit, but also causes undeveloped stamens. In agreement, *SlIAA9* mRNA is visible in stamen floral meristem, in emerging stamens and during subsequent development as well as in other floral organs (Wang et al., 2005). Similarly *SlTIR1*, homologous to Arabidopsis *AtTIR1*, is expressed in stamens in all developmental stages before anthesis (Ren et al., 2011). *SlFLOOZY* (*ToFZY*), the putative tomato orthologous gene of *AtYUC4*, has been identified and it is expressed in immature flowers. In addition tomato genome contains many genes that encode flavin monooxygenases-like proteins, similar to the situation observed for Arabidopsis *YUC* genes (Expósito-Rodríguez et al., 2007). This data suggest that most genes involved in auxin biosynthesis and signaling are conserved in tomato.

Indirect evidence on the role of auxin transport in tomato stamen formation comes from the culture of tomato apices on NPA-containing medium that results in naked *pin1*-like inflorescences (Reinhardt et al., 2000). Among the 10 homologs of Arabidopsis *PIN* genes (*SlPIN1-10*) found in tomato, *SlPIN4* is the most expressed, although at a low level, in stamens during flower development up to the anthesis. In agreement P_35S_:*SlPIN4*^RNAi^ flowers show alterations in stamen morphogenesis and development together with abnormal sepals and parthenocarpic fruit development (Mounet et al., 2012). This evidence suggests that SlPIN4 might be the major PIN player in regulating both stamen morphogenesis and development. An additional role for auxin transport during early and late stamen development has been suggested by phenotypical analysis of *polycotyledon* mutants (*poc*) (Madishetty et al., 2006). The *poc* loss-of-function mutation causes an increase in the basipetal auxin transport in stems and due to several abnormalities in floral organs, possibly in flowers. In particular, *poc* mutants have an increased number of stamens characterized by the lack of the typical fusion of filaments. Late developmental processes are also altered, since *poc* anthers lack dehiscence and stamens are shorter, possibly due a reduction in epidermal cells length (Al-Hammadi et al., 2003). Future studies will be necessary to confirm and expand this evidence that suggest a fundamental role for PAT in early and late stamen development.

Medicago (*Medicago truncatula*) is a model species for the study of flower morphogenesis in legumes. The main difference in Medicago stamen development compared to Arabidopsis is the existence of four common primordia from which petals and stamens differentiate. Medicago flowers are organized in four whorls and show a pentamerous arrangement of sepals and petals, ten stamens and a central carpel. Nine stamen filaments are joined in a staminal tube around the carpel, and the tenth, the vexillary stamen filament at the adaxial position, is free standing.

Medicago *YUC*-like genes *MtYUC1*, *MtYUC2*, and *MtYUC3* have been found by sequence homology search but no functional data has been reported yet (Tivendale et al., 2010). However, a gradient of DR5 activity with a maximum of auxin at the tips of floral organs has been detected and is required for proper development of primordia (Zhou et al., 2011).

Medicago has more *PIN* and *LAX* genes than Arabidopsis (8 *PINs* and 4 *AUX/LAXs* in Arabidopsis vs. 9 *PINs* and 5 *LAXs* in Medicago) (Schnabel and Frugoli, 2004). Phylogenetic analysis indicated that *MtPIN4*, *MtPIN5*, and *MtPIN10* belong to a small cluster and are all homologous to the Arabidopsis *PIN1*. MtPIN10, which shows 65% amino acid identity with AtPIN1, indeed seems to have a role in stamen development but, in contrast to Arabidopsis *pin1* mutants, *mtpin10* mutants develop flowers. However these flowers have unfused stamens, also reduced in number, together with altered sepals and carpels and are sterile. This data suggests a role for PAT, and in particular for MtPIN10, in stamen (and other floral organs) primordia formation and separation (Peng and Chen, 2011; Zhou et al., 2011).

### Monocots

The Poaceae (grasses) family, one of the largest flowering plant families in angiosperms, includes many economically important crops such as maize and rice. The grass inflorescence is composed of different types of branches, including a specialized branch called spikelet. The spikelet is a special unit of the inflorescence and forms one to several florets, depending on the species. In the floret, floral organs such as perianth organs, carpels, and stamens are formed. In grasses, different types of meristem, such as the inflorescence meristem (IM), the branch meristem (BM), the spikelet pair meristem (SPM, present only in some grasses), the spikelet meristem (SM) and the floral meristem (FM), are responsible for the complex development of inflorescences and flowers.

Maize (*Zea Mays*) is monoecious and the male inflorescence, the tassel, situated at the shoot apex, consists of a main spike with several long lateral branches at the base. Normal tassels produce florets whose meristems give rise to a lemma and palea (outer whorl structures derived from bracts or sepals), two lodicules (derived from petals), and three stamens.

Auxin has a main role in maize stamen primordia formation as the single mutant *spi1*, defective in a flavin monooxygenase with similarity to the *YUC* genes, show a reduced number of stamens. In addition, auxin maxima (visualized by DR5:RFP), are clearly associated with the initiation of floral organ primordia, including stamen primordia in tassel (Gallavotti et al., 2008a).

A role for auxin transport in stamen primordia formation is suggested by the phenotype of the mutant *bif2*, which is defective in a serine/ threonine protein kinase co-orthologous to *PID*, and which has florets with no floral organs at all, or reduced stamen numbers. In addition, when stamens arise, they are deformed, suggesting that *bif2* can be also involved in the subsequent stamen developmental phases (McSteen and Hake, 2001). Similar to the mutation in *bif2*, *Bif1*, a classical semidominant mutation of maize, causes fewer florets and a reduced number of stamens in most florets. Many of the phenotypes seen in *Bif1* (and *bif2*) mutants are also seen in plants treated with auxin transport inhibitors (Wu and McSteen, 2007) and, consistently, *Bif1* mutation causes a reduction of auxin transport. This data suggests a role for *Bif1* in regulating auxin transport (Barazesh and McSteen, 2008). BIF2 has been shown to be able to phosphorylate ZmPIN1a, one out of the three homologs of *PIN1* in maize, called Zm*PIN1*a, -b and -c. ZmPIN1a driven by the *AtPIN1* promoter can rescue most, but not all, defects of the Arabidopsis *pin1-3* mutant, as some flowers still show the lack of stamens. This data suggests an at least partial conservation in polar auxin transport mechanisms between maize and Arabidopsis (Gallavotti et al., 2008b). Basal ZmPINs (ZmPIN1a, ZmPIN1b, and ZmPIN1c) localization has been shown in different plant organ tissues such as coleoptile cells (Nishimura and Koshiba, 2010). ZmPIN1s seem to be localized in the L1/epidermal outer layer and in the inner tissues of male inflorescence meristem, according to Carraro et al. (2006), although this data has not been confirmed by subsequent analysis (Forestan and Varotto, 2012). However its localization during stamen primordia formation still needs to be determined. Thus, further experiments are necessary to shed further light on the role of auxin transport in stamen formation. Furthermore, very recently a previously uncharacterized ZmPIN protein most closely related to PIN1 that is present in all flowering plants but lost in the Brassicaceae, including Arabidopsis, has been identified in maize, called Sister-of-PIN1 (SoPIN1). *SoPIN1* expression is evident in the L1 and L2 layers of spikelet meristems (O'Connor et al., 2014). In addition one more member of *PIN1* cluster (named *ZmPIN1d*), one gene homologous to *AtPIN2* (*ZmPIN2*), three orthologs of *PIN5* (*ZmPIN5*a–c), one gene paired with *AtPIN8* (*ZmPIN8*), and three monocot-specific *PINs* (*ZmPIN9*, *ZmPIN10a*, and *ZmPIN10b*), 2 *ABCB*-like genes, *ZmABCB1/BR2* and three orthologs of *AtABCB19* (*ZmABCB2*, *ZmABCB10_1*, and *ZmABCB10*_*2*) have recently been identified (Knöller et al., 2010; Forestan and Varotto, 2012; Forestan et al., 2012).

In conclusion, it is not possible to infer from the available data to what extent auxin transport is involved in stamen primordia formation/development in maize. Future experiments should focus on the identification of all players that can contribute to stamen morphology and growth in order to propose a unified model of stamen development.

Rice (*Oryza sativa*) inflorescence architecture is quite different from that of maize, as flowers are hermaphrodite, and one rice spikelet has only one floret surrounded by a pair of empty glumes (corresponding to sepals). In addition, rice florets have an asymmetric structure with five types of floral organs: one lemma and one palea in the first outer whorl, two lodicules in the second whorl, six stamens in the third whorl and one pistil with two stigmas in the fourth innermost whorl.

At least seven *YUC*-like genes have been found in rice, and it is very likely that these *OsYUCs* are also redundantly involved in IAA biosynthesis in different organs. *OsYUC1* is the gene most closely related to *AtYUC1* and *AtYUC4* and is the ortholog of Zm*SPI1*. Although *OsYUC1* is expressed in developing flowers, a phenotypical analysis of flowers from the available *OsYUC1* antisense or overexpressing plants is still lacking (Yamamoto et al., 2007). However, the fact that *tdd1* plants, defective in a protein which catalyses the first step of the Trp biosynthesis pathway, show reduced IAA content and flowers with altered stamen number and sometimes malformed stamens, suggests that auxin—and, possibly, auxin transport—controls stamen development in rice as well (Sazuka et al., 2009). Indeed, overexpression of the single rice *PID* ortholog, *OsPID*, alters the number of floral organs and leads to a reduction of stamen number and an increase of the number of stigmas. In addition, developing stamens show an overall abnormal morphology, a phenotype that could be phenocopied by treatment with NPA (Morita and Kyozuka, 2007). Rice has also three PIN1 orthologs (*OsPIN1*, *OsPIN1b*, and *OsPIN1c*). Functional analysis has been reported only for *OsPIN1* and revealed that *OsPIN1* knockdown plants do not have the characteristic *pin* phenotype. However, *OsPIN1* is highly expressed in flowers, and in particular in stamen filaments and at the junction region between anther and filament during late development (Xu et al., 2005). Similarly *OsPIN3t*, which shows high aminoacid sequence identity to *AtPIN3*, is expressed in mature anthers (Zhang et al., 2012). Phenotypic analysis of double and triple *OsPIN1* mutant flowers could shed light on the role of these transporters in stamen development.

Taken together, this data suggest, but are far from being conclusive, a role for auxin transport in stamen primordia formation such as in maize, and also in stamen filament elongation during late flower development.

## Conclusions

The data reported in this review, (summarized in Table 1) clearly show the fundamental role of PAT in the formation of stamen primordia in Arabidopsis. The general model proposed by Benková et al. (2003) on floral organ primordia formation, and supported by van Mourik et al. (2012) for stamens, is mainly based on the direction of auxin flow mediated by PIN1 that creates the auxin maxima in the L1 layer—at stage 5 of flower development—and an auxin gradient in the subsequent stages due to basipetal auxin transport. The integrated model is presented in Figures 3A,B. However, additional work is necessary to assess PIN3 and PIN7 role in stamen primordia formation and to establish the contribution of other efflux carriers such as ABCB/PGP proteins as well as that of influx carriers. This will shed light also on the direction of auxin transport during subsequent developmental stages leading to stamen morphogenesis.

Auxin transport also appears to regulate later developmental processes that require coordination with each other and with gynoecium development. Basipetal auxin transport—exerted by different members of large-loop PINs and ABCB/PGP families—through epidermal and vascular cells regulates stamen filament elongation is described in the model presented in Figure 3C. However, whether other auxin transporters are involved in filament elongation and whether PAT coordinates late development of male and female reproductive organs remains to be investigated. Current data also indicate that different ER-localized PINs, which affect auxin concentration, regulate pollen development and germination. However the role of large-loop PINs as well as that of ABCB proteins in regulating the timing of anther dehiscence in these latter processes is still elusive.

The mechanisms of auxin transport, together with those of auxin biosynthesis and signal transduction, appear conserved in dicots and monocots. Alignment of *AtPIN1* and other dicot-specific orthologs, with monocot members of the *PIN1* family indicates that all *PIN1* genes share a common overall structure. However the monocot *PIN* family is wider and more divergent than the dicot one, with three or four genes homologous to one single Arabidopsis *PIN* gene. In addition, the overall expression profile of the *PIN* genes is different between Arabidopsis and maize: while in Arabidopsis each *PIN* has a specific expression domain, many *ZmPIN* genes are simultaneously expressed in the same organ or tissue (Forestan et al., 2012). In addition, *PID*-like genes have been also identified in maize and rice (McSteen and Hake, 2001; Morita and Kyozuka, 2007). Furthermore, orthologs of *AtABCB1/PGP1* and *AtABCB19/PGP19* have also been identified in maize (Knöller et al., 2010), sorghum and rice (Shen et al., 2010) but no data is currently available on their role in flower and stamen development.

In summary, the available data suggests that auxin transport-dependent mechanisms are required for proper stamen development and in particular for stamen primordia formation in dicots as well as in monocots. However to propose a model of more general validity all actors need to be identified to gain a full understanding of how the auxin gradient during stamen development is established. Furthermore more work directly focused on stamen growth is necessary to understand the involvement of PAT in different processes of early and late stamen development.

### Conflict of interest statement

The authors declare that the research was conducted in the absence of any commercial or financial relationships that could be construed as a potential conflict of interest.
